# The RAAS Goodfellas in Cardiovascular System

**DOI:** 10.3390/jcm12216873

**Published:** 2023-10-31

**Authors:** Ilaria Caputo, Giovanni Bertoldi, Giulia Driussi, Martina Cacciapuoti, Lorenzo A. Calò

**Affiliations:** Nephrology, Dialysis and Transplantation Unit, Department of Medicine—DIMED, University of Padua, Via Giustiniani, 2, 35128 Padova, Italy; ilaria.caputo@unipd.it (I.C.); giovanni.bertoldi@unipd.it (G.B.); giulia.driussi@aopd.veneto.it (G.D.); martina.cacciapuoti@aopd.veneto.it (M.C.)

**Keywords:** RAAS, Ang 1-7, Ang 1-9, cardiovascular remodeling, oxidative stress

## Abstract

In the last two decades, the study of the renin–angiotensin–aldosterone system (RAAS) has revealed a counterregulatory protective axis. This protective arm is characterized by ACE2/Ang 1-7/MasR and Ang 1-9 that largely counteracts the classic arm of the RAAS mediated by ACE/Ang II/AT1R/aldosterone and plays an important role in the prevention of inflammation, oxidative stress, hypertension, and cardiovascular remodeling. A growing body of evidence suggests that enhancement of this counterregulatory arm of RAAS represents an important therapeutic approach to facing cardiovascular comorbidities. In this review, we provide an overview of the beneficial effects of ACE2, Ang 1-7/MasR, and Ang 1-9 in the context of oxidative stress, vascular dysfunction, and organ damage.

## 1. Introduction

**Figure 1:**
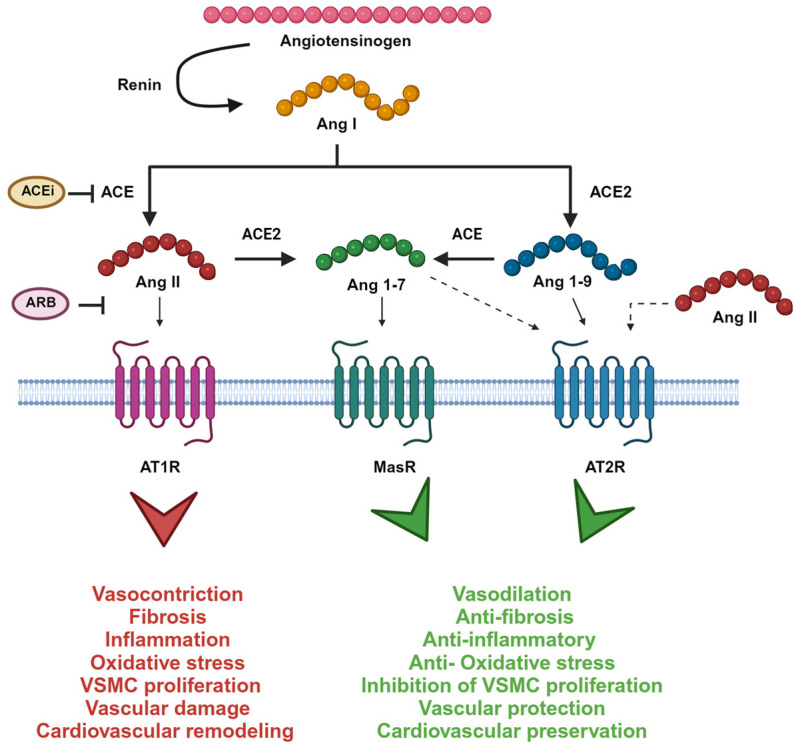
The renin–angiotensin system enzymatic cascade. Ang I is cleaved by angiotensin-converting enzyme (ACE) to Ang II, which is metabolized by ACE2 to Ang 1-7. Ang II binds to Ang II type 1 receptor (AT1R) inducing pro-hypertensive effects. Ang 1-7, instead, binds to Mas receptor (MasR) and opposes Ang II/AT1R actions. Ang I can also be metabolized to Ang 1-9 by ACE2. Ang 1-9 recognize Ang II type 2 receptor (AT2R) inducing a protective effect as well as Ang 1-7. Also, Ang II recognizes AT2R but with a lower affinity. Picture created with BioRender.com.

The main pathway involved in the regulation of physiology and homeostasis in the cardiovascular system is the renin–angiotensin–aldosterone system (RAAS). RAAS is responsible for the maintenance of vascular tone by regulating extracellular fluid volume and blood pressure [1]. In addition to a systemic RAAS playing an endocrine role in the human body, the concept of a local RAAS was also demonstrated, having autocrine, paracrine and intracrine functions. Indeed, even locally, RAAS components can be produced and be active, limited to tissues or organs such as kidney, heart, vessels, adipose tissue, lymphatic tissue, central nervous system, adrenal and pituitary gland, reproductive tissues and gastrointestinal tract, hematopoietic tissues [2,3,4]. Nowadays, it is currently accepted the existence of two different branches of RAAS: the classic one, ACE/AngII/AT1R/aldosterone, and the counterregulatory arm ACE2/Ang 1-7/MasR axis, which is known to counterbalance the former. Unbalanced RAAS give rise to cardiovascular and renal diseases like hypertension, but also congestive heart failure, obesity, hepatic complications, diabetes, neuronal disease, and miscarriage, endothelialitis, fibrosis, atherosclerosis [1] (Figure 1). 

Renin is a proteolytic enzyme synthesized in its inactive form (pro-renin) by the juxtaglomerular cells of the kidney. In response to reduced distal tubular delivery of sodium, reduced pressure in the afferent arterioles of the glomerulus, and sympathetic activation, pro-renin is released into the bloodstream where it is converted into active renin, responsible for the conversion of angiotensinogen into the decapeptide angiotensin (Ang) I [1,5]. Angiotensinogen is an inactive decapeptide mainly produced by hepatocytes, but production within other cell types may contribute to local and circulating concentrations of Ang II in pathophysiological conditions [6]. Plasma levels of angiotensinogen might increase in response to corticosteroid, estrogen, thyroid hormone stimulation, as well as to angiotensin (Ang) II [7]. Angiotensin-converting enzyme (ACE) hydrolyzes Ang I into Ang II.

ACE plays an important role also in the kallikrein–kinin system (KKS) as it is the major intravascular peptidase of BK, producing des-Arg9-BK and several inactive intermediates [8]. BK is a potent vasodilator that contributes to hypotension by increasing vascular permeability, plasma extravasation, and bronchoconstriction [9].

Ang II is the main player of RAAS. It is active in different tissues and cell types as endothelial cells, vascular smooth muscle cells (VSMCs), and epithelial cells, it has been found at high levels in hypertensive patients [10] and it acts mainly via two different receptors: angiotensin type 1 receptor (AT1R) and angiotensin type 2 receptor (AT2R). AT1R is involved in hypertension, pro-inflammatory response, vasoconstriction, increased heart rate, increased production of ROS species, the release of prostaglandins, and an increase in intracellular calcium concentration [1,11]. AT2R plays instead a protective role, inducing anti-inflammatory, anti-fibrotic, and anti-proliferative effects, vasodilation and therefore counterbalancing the effect mediated by AT1R [11]. Both Ang II receptors are widely expressed in the lung, particularly in epithelial cells, VSMCs, and macrophages, and are members of the seven transmembrane—domain G protein-coupled receptor (GPCR) superfamily [3,11]. BK receptors are known to heterodimerize with angiotensin receptors AT1R, AT2R, and Mas which may augment or diminish their activity [8]. Ang II induces hypertension not only by playing a role in vasoconstriction but also through sodium and water handling: by activating sodium transporters in the proximal tubules and increasing blood pressure, sodium excess is excreted. Moreover, Ang II induces aldosterone production by the zona glomerulosa of the adrenal cortex. Aldosterone is a steroid hormone whose effects are mediated by the mineralocorticoid receptors (MRs) which act as a transcription factor for several genes involved in the regulation of blood pressure and sodium–potassium balance [12]. Abnormalities in aldosterone synthesis can be due mainly to aldosterone-producing adenoma or to genetic mutations (familial hyperaldosteronism) [13] leading to a pathological condition known as primary aldosteronism which is associated with hypertension, hypokalemia, and metabolic syndrome, increasing cardiovascular risks [12,13,14].

Blockade of the regulatory branch of RAAS is of particular interest in the development of new pharmacological therapies. Four different classes of antihypertensive drugs are clinically used: ACE inhibitors (ACEis), angiotensin type 1 receptor antagonists (ARBs), renin inhibitors, and mineralocorticoid receptor antagonists (MRA).

**Figure 2:**
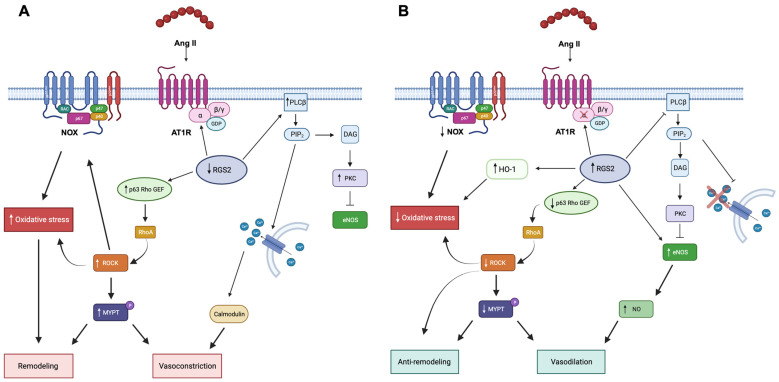
Ang II signaling in cardiovascular remodeling. Panel (**A**) Angiotensin II in hypertensive patients is promoted through oxidative stress vasoconstriction, vascular remodeling, and insulin resistance. Ang II, angiotensin II; AT1R, angiotensin II receptor 1; PLC β, phospholipase C β; PIP2, phosphatidylinositol diphosphate; DAG, diacylglycerol; PKC, protein kinase C; eNOS, endothelial nitric oxidase; NOX, NAD(P)H oxidase, ROCK, Rho Kinase; MYPT, myosin phosphatase protein target. Panel (**B**). Angiotensin II signaling in BS/GS is blunted at post-receptor level, thereby promoting vasodilation and insulin sensitivity through RGS-2 activity [19]. RGS, regulators of G-protein signaling; HO-1, heme-oxygenase 1; NO, nitric oxide. Picture created with BioRender.com.

Patients affected by Gitelman syndrome (GS) or Bartter syndrome (BS) are characterized by mutations in genes coding for ionic transporters which lead to altered sodium reabsorption and electrolyte balance, hypokalemia, and metabolic alkalosis [15]. Interestingly, these patients display a high activation of their RAAS without following hypertension: indeed, despite the high levels of Ang II and aldosterone, BS/GS patients are normotensive or hypotensive [16,17]. Extensive studies from our laboratory have shown that BS/GS patients are a unique human model of AT1R endogenous antagonism. Indeed, although in BS/GS Ang II receptors expression and affinity are normal and RAAS is activated with high levels of Ang II, these patients display peculiar clinical signs of an abnormal regulation of the Ang II-mediated signaling cascade [15,18] which is blunted compared to hypertensive patients (Figure 2).

## 2. ACE2/Ang 1-7/MasR Axis and Ang 1-9

The counterregulatory effects of RAAS are mediated by the ACE2/Ang 1-7/MasR axis. Indeed, all these molecules play a protective role in several cardiovascular diseases, and, in experimental disease models, Ang 1-7/MasR signaling suppressed Ang II-induced effects [20]. Also Ang 1-9 seems to play a very important role in protection from cardiovascular remodeling [21].

ACE2 is a carboxypeptidase mainly involved in the degradation of Ang I into Ang 1-9 and of Ang II into Ang 1-7, making this enzyme likely more important than ACE in regulating local levels of Ang II and Ang 1-7, and therein the balance of RAAS activation [22]. In addition to its role as a player of RAAS, ACE2 is also well known to be the viral receptor for some coronaviruses, including SARS-CoV-2 which recently caused the COVID-19 pandemic. Ang II was shown to increase ACE2 and SARS-CoV-2 infection in a human model of bronchial epithelial cells, while treatment with the ARB irbesartan blunted this effect [23]. Indeed, high Ang II levels were associated with a worsening of COVID-19 condition and a risk factor for infection, while treatment with RAAS inhibitors played a neutral or beneficial role in these patients [24,25,26,27,28]. However, despite the high levels of Ang II, BS/GS patients resulted to be protected by COVID-19 likely due to their characteristic metabolic alkalosis. Indeed, SARS-CoV-2 binds to the glycosylated form of ACE2 and requires the enzymatic activity of peptidases like cathepsin-L to enter the host cell. The metabolic alkalosis induced altered intracellular pH in BS/GS patients, which interferes with the glycosylation of the viral receptor ACE2 and cathepsin-L activity, therefore representing a protective mechanism against SARS-CoV-2 infection [29,30]. ACE2 was first discovered in 2000 by Tipnis and Donoghue [31,32]. Experiments performed in ACE2 knockout mice models underline the importance of this enzyme in diabetes [33], cardiac function, hypoxia, cell contractility [34,35,36], endothelial function [37], liver [38], and kidney injury [39,40]. Its overexpression is associated with improved pressure and glycemic control, reduced oxidative stress and modulation of ER stress [41], prevention of cardiac hypertrophy and fibrosis induced by Ang II [42], and of acute lung injury by the modulation of pro-inflammatory molecules [43], while its loss leads to cardiac dysfunction, hypertrophy, fibrosis and a greater diastolic function [44]. Moreover, few studies reported the role of *ACE2* gene polymorphisms related to hypertension and cardiovascular risk. Several studies are available on the Chinese population, reporting different ACE2 variants associated with the induction of essential hypertension [45]. Among these, rs2106809 variant with TT genotype and rs2074192 T allele in *ACE2* gene has been associated with lower Ang 1-7 levels in women, likely downregulating ACE2 expression/activity and therefore increasing the risk of hypertension [46,47]. In addition, two independent studies on Chinese [48] and German [49] populations reported that minor alleles of ACE2 single nucleotide polymorphisms (SNPs) were associated in men, with hypertrophic cardiomyopathy and left ventricular hypertrophy, respectively. In broader studies, the variant rs2285666 with the AG genotype has been related to a lower risk of developing hypertension in women, while the AA genotype was related to a higher risk of hypertension in different ethnic populations [50]. The A allele of these polymorphisms influences the risk of cardiovascular events in women, as reported by the results of the MORGAM study in 2011 [51]. This study has also underlined the importance of gender and ethnic group differences.

The protective role of ACE2 is due mainly to the action of Ang 1-7. In addition to ACE2, neprilysin (NEP) as well as prolyl endopeptidases and thimet oligopeptidases can participate in Ang 1-7 biosynthesis, depending on tissue or cell type [41,52]. Ang 1-7 is a heptapeptide well known to counterbalance the effect of Ang II through the activation of its receptor, Mas, which is a G-protein coupled receptor whose signal leads to vasorelaxation, anti-inflammatory, anti-fibrotic, and anti-proliferative effects. Through its binding to the Mas receptor (MasR), Ang 1-7 improves insulin sensitivity and glucose tolerance in experimental animal models [41]. It also stimulates NO synthase (NOS) in endothelial cells and the production of nitric oxide (NO) in platelets, preventing platelet activation and coagulation, as well as in VSMCs and cardiac cells, influencing vascular tone and cardiac contraction. Moreover, it promotes bradykinin production which, though bradykinin receptor type 2 (BK2R), leads to NO formation and it can antagonize AT1R-mediated activation of MAP kinases, key regulators in several cell signaling pathways and is involved in vascular remodeling, renal fibrosis, and cardiac hypertrophy [41]. Ang 1-7 also attenuates LPS-induced pulmonary fibrosis down-modulating AT1R and increasing MasR expression [53] and displays cardioprotective effects in heart failure conditions [54]. Since MasR, as GPCR can heterodimerize with receptor subtypes of other GPCRs, it may interact both with AT1R and AT2R, as it does with BK2R-inducing vasorelaxation [20]. In addition to MasR, Ang 1-7 can also bind the AT2R and, via this receptor, it prevents aneurysmal rupture and plays a vasoprotective and atheroprotective role in experimental models of atherosclerosis [20]. Further evidence of the beneficial effects of the ACE2/Ang1-7/MasR axis was reported by Abuohashish [55], where inhibition of ACE by captopril led to an upregulation of this branch of RAAS, improving bone metabolism, mineralization, and mass and reduced RANKL expression which can be increased by Ang II stimulation. Also, Savoia and coworkers [56] reported that, in an in vivo model of spontaneous hypertensive rats (SHR), chronic AT1R blockade enhances expression and activation of MasR, contributing to improved vascular remodeling, and this effect is associated with reduced ROS production, increased NO bioavailability and it is independent on AT2R.

In addition to Ang 1-7, ACE2 is also able to produce Ang 1-9 whose role is still not well defined, but a growing body of evidence reports the beneficial role of this molecule in hypertension and cardiovascular remodeling. Ocaranza and coworkers [57] were the first to show an independent role of Ang 1-9, until then considered an inactive peptide, whose beneficial role was due to the competition for ACE binding site with Ang I, limiting the new synthesis of Ang II. In this study, they induced myocardial infarction (MI) by coronary artery ligation and observed an increase in Ang II, Ang 1-9, ACE2, and ACE levels after 1 week of MI, while only ACE2 and Ang 1-9 levels decreased after 8 weeks. Importantly, when rats were treated with the ACEi enalapril, plasma Ang 1-9 levels were significantly high both in MI and sham-operated rats at 8 weeks while Ang 1-7 levels did not change in any phase after MI [57]. Another important evidence by Flores-Munos [58] reported that Ang 1-9 specifically binds to AT2R, antagonizing the Ang II signaling in cardiomyocytes and underlining the role of this peptide in the protective branch of RAAS. Nowadays, it is well known that Ang 1-9 plays an anti-hypertensive and anti-remodeling role by inducing vasodilation, improving renal function, increasing eNOS levels and NO production, and lowering inflammation [59]. An overview of ACE2, Ang 1-7/MasR, and Ang 1-9 effects is provided below.

### 2.1. Oxidative Stress

**Figure 3:**
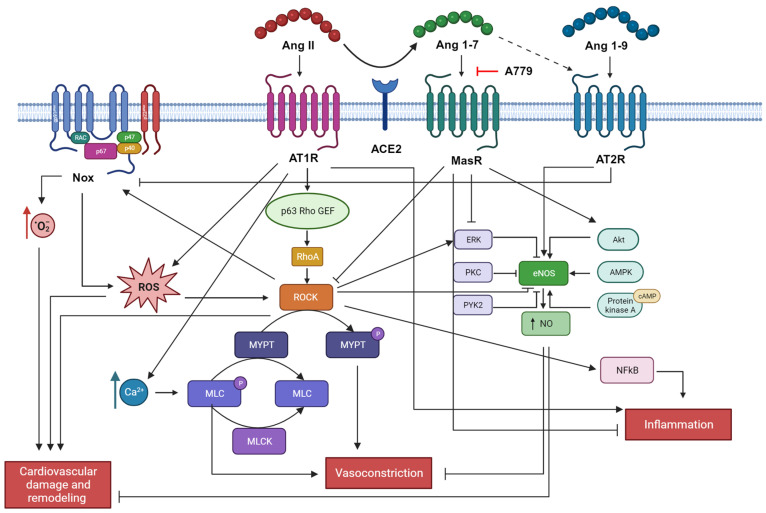
Ang II signaling in cardiovascular remodeling. Ang II induces cardiovascular damage and remodeling, vasoconstriction, and inflammation through the enhancement of oxidative stress, contractile machinery, pro-inflammatory cytokines release, and blockade of eNOS. On the contrary, Ang 1-7 and Ang 1-9 counteract these actions sustaining an anti-oxidant environment and vasodilation. Picture created with BioRender.com.

Oxidative stress is defined as an imbalance between the production of reactive oxygen species (ROS) and antioxidant defenses. ROS usually refers to free radicals (such as superoxide O_2_^−^, hydroxyl radical OH^−^, nitric oxide NO) or non-radical intermediates (like hydrogen peroxide H_2_O_2_, peroxynitrite ONOO^−^) able to react with surrounding molecules, playing a messenger role in several biochemical cascades involving lipid and protein metabolism, DNA damage and, macroscopically, an increase in ROS is usually associated with an increase in cardiovascular damage and remodeling [60,61] (Figure 3). The major sources of ROS are the nicotinamide adenine dinucleotide phosphate (NADPH) oxidases (Nox), multi-component enzymes that catalyze electron transfer from NADPH to O_2_ [62]. Seven isoforms of Nox are currently known: Nox1, Nox2, Nox3, Nox4, Nox5, Duox1, and Duox2 and are widely expressed in cardiovascular and renal tissues [60,61]. The general structure of a Nox comprises an enzymatic core consisting of two integral membrane proteins gp91^phox^ and p22^phox^, and regulatory subunits including cytosolic proteins p47, p67, and p40 and GTPase Rac (Rac1 or Rac2) [62]. Another important enzyme involved in the generation of ROS is nitric oxide synthase (NOS). NO plays a pleiotropic role in the cardiovascular system and its production is dependent on NOS activity, which requires the substrate L-arginine and the cofactor tetrahydrobiopterin (BH_4_). Without these factors or with an oxidized BH_4_, NOS is in an uncoupled form, leading to the production of O_2_^−^ and peroxynitrite [61,63]. Three isoforms of NOS have been identified, each one encoded by a distinct gene: brain or neuronal NOS (nNOS; encoded by *NOS1*), inducible NOS (iNOS; encoded by *NOS2*), and endothelial NOS (eNOS; encoded by *NOS3*), the most common form in the cardiovascular system [64]. In humans, eNOS is activated by phosphorylation at the Ser1177 by AKT and dephosphorylation at the Thr495 by protein phosphatase (PP) 1 and PP2A and calcineurin (or PP2B) [64]. Also, 5′-AMP-activated protein kinase (AMPK), calcium/calmodulin-dependent protein kinase type II (CaMKII), and cAMP-dependent protein kinase (also known as protein kinase A; PKA) are able to phosphorylate eNOS at Ser1177, activating its enzymatic activity. On the contrary, phosphorylation at Ser114 by extracellular signal-regulated kinase 1 (ERK1) and/or ERK2 and PKC as well as at Tyr657 by protein tyrosine kinase 2β (PYK2) is known to decrease NO synthesis [64].

Ang 1-7 modulates mitogen-activated protein kinases (MAPKs) (ERK, p38, JNK), by reducing their activity [65,66]. Moreover, the RhoA/Rho-kinase signaling pathway is involved in the regulation of eNOS activity as well as vascular contraction through Ca^2+^ sensitization [67,68,69]. Indeed, agonist binding to G-protein-coupled receptors (like Ang II [67]) induces Rho-kinase activity through GEF activation and binding to the active GTP-bound RhoA. Among the target molecules of Rho-kinase activity, there are myosin light chain (MLC) (essential for VSMC contraction) myosin phosphatase target subunit (MYPT)-1, ezrin/radixin/moesin family, adducin, phosphatase and tensin homolog, eNOS, Tau and LIM-kinase [68]. Also, ROS are able to activate the RhoA/Rho-kinase signaling pathway [70].

Ang 1-7, through Mas, stimulates eNOS activation and NO production via Akt-dependent pathways, thus playing an antioxidative role in the cardiovascular system [65,71]. Using Chinese hamster ovary (CHO) cell lines and human aortic endothelial cells (HAECs) Oliveira Sampaio and coworkers demonstrated that Ang 1-7 strongly increases NO production in a dose-dependent manner, regulates Ser1177/Thr495 phosphorylation of eNOS and increases the phosphorylation of Akt. Using A779, a specific inhibitor of the Mas receptor, all these events were reversed, suggesting that the Ang 1-7 effects are mediated by Mas. Inhibition of the PIK3/Akt pathway using Wortmannin, results in inhibition of the Ang 1-7 mediated effects as well, thus providing evidence that the antioxidative role of Ang 1-7 through the Mas is Akt-mediated [71]. Ang 1-7 was also found to attenuate atherosclerosis through a NO-mediated mechanism in apolipoprotein E knockout (ApoE-KO) mice [72].

An in vivo study using *Mas* gene-deleted mice on a pure genetic background (Mas^−/−^) reported an increase in blood pressure, a decreased eNOS expression in the aorta, and a reduced urinary excretion of nitrite and nitrate, suggesting a reduced NO bioavailability overall. Moreover, malondialdehyde (MDA), a marker of oxidative stress, was found at high levels in Mas^−/−^ mice compared to controls as well as the catalytic subunit of Nox2 gp91^phox^ protein content [73]. Activation of the PI3K/Akt/eNOS pathway also increases levels of guanosine 3′,5′-cyclic monophosphate (cGMP) in blood vessels [65]. In a model of rat primary fibroblasts, Ang 1-7, through its Mas receptor, reduced fibrosis by modulating the activation of the TGFβ/NOX4/ROS/RhoA/Rock pathway [74]. Inhibition of the ROCK pathway with Fasudil was associated with a decrease in blood pressure levels and pMYPT1/MYPT1 ratio in DOCA hypertensive rats [75]. In the same study, eNOS mRNA levels and Ang 1-9 plasma levels were found to be increased after Fasudil treatment in the hypertensive animals, together with a decrease in the TGF-β1, PAI-1, and MCP-1 mRNA and protein levels, all molecules associated with aortic wall remodeling. Moreover, ACE2 gene expression and activity were importantly increased after ROCK inhibition, while ACE gene expression and activity together with Ang II plasma levels were reduced [75]. In a cellular model of human umbilical artery smooth muscle cells (HUASMCs), it was demonstrated that pretreatment with recombinant hrACE2 prevented the Ang II-induced JAK2–STAT3–SOCS3 and reduced superoxide generation and MDA levels, thus providing evidence that ACE2 strongly blunts oxidative stress [76].

Exploring the role of Ang 1-9 in the oxidative stress pathway, it was found that infusion of Ang 1-9 increased eNOS mRNA expression and plasma nitrate levels in hypertensive rats, also reducing cardiac and aortic O_2_^−^ production and NADPH oxidase activity in the same model [77].

Several studies from our group reported a strong activation of the ACE2/Ang 1-7/MasR axis in BS/GS patients, coupled with a reduced expression of proteins involved in the oxidative stress (as p22^phox^), reduced activation of the RhoA/Rho-kinase pathways, increase NO production and endothelial protection [15,18,19,78]. A comparative study between normotensive, hypertensive, and BS/GS patients reported that the p63RhoGEF, both at mRNA and protein level and MYPT-1 phosphorylation status were higher in hypertensive patients and lower in BS/GS patients compared to controls [79] (Figure 2). Also, in Fabry disease patients, a genetic lysosomal storage disorder, we have reported the important role of oxidative stress, which contributed to the cardiovascular–renal remodeling of these patients [80]. In addition, we have also shown evidence that green tea administration (as adjuvant antioxidant treatment) on top of enzymatic replacement treatment (ERT) reduced oxidative stress in terms of ROCK activation, reducing p22^phox^, ERK1/2, and MYPT-1phosphorylation which is coupled with increased eNOS activity and NO production, to improve endothelial cells’ integrity [81].

### 2.2. Endothelial and Vascular Damage

Vasculature is the result of layers of different cells (endothelial, smooth muscle, and fibroblasts) interacting with each other, able to respond promptly to microenvironmental changes (both biochemical or physical) through local production of mediators that influence their structure and function [3,82]. Vascular damage is usually associated with impaired endothelial function, alteration in VSMC contraction/relaxation processes, cellular migration, growth, cell death, production or degradation of ECM, and inflammation [3,82,83].

Ang II is a well-known trigger of cardiovascular remodeling [3,66,74,84].

Interestingly, as mentioned above, even with activation of RAAS and high levels of Ang II, BS/GS patients present downregulation of oxidative stress, reduced vascular tone, and absence of cardiovascular remodeling [16] together with high levels of Ang 1-7 and ACE2 [85], suggesting an overactivation of the ACE2/Ang 1-7/MasR axis, representing a unique human model of Ang II antagonism (Figure 2).

Ang 1-7 is able to counteract the pro-fibrotic effect mediated by Ang II [66,74,86]. There is also evidence that Ang II-induced profilin-1–MAPK signaling and administration of recombinant hrACE2 prevented it from a cellular model of human umbilical artery smooth muscle cells (HUASMCs), suggesting that ACE2 plays an anti-proliferative role in this cell type [76]. Overexpression of ACE2 in a model of endothelial cells treated with Ang II in vitro strongly reduced the production of adhesion molecules and inflammatory cytokines like MCP-1, VCAM-1, and E-selectin and pro-oxidant and pro-inflammatory environment induced by Ang II, resulting overall in a better endothelial function, which is reflected by impairment of early atherosclerotic lesions in the in vivo model of ApoE-deficient mice and Ang 1-7 enhance the effects mediated by ACE2 overexpression [87]. ACE2 deletion, on the contrary, resulted in vascular dysfunction, reduced NO production through an Akt-independent mechanism, and increased oxidative stress, further underlining the protective role of this molecule in cardiovascular remodeling [88]. Moreover, ACE2 deficiency in ApoE-KO mice results in an accelerated atherosclerotic process, increased vascular inflammation with high levels of IL-6 and TNFα, increased expression of adhesion molecules, and high responsiveness to inflammatory stimuli [89].

In an in vivo model of Mas^−/−^ mice, it was evaluated the vascular reactivity and endothelial function by measuring changes in mean arterial pressure in response to administration of the endothelium-dependent vasodilator acetylcholine (Ach) or sodium nitroprusside (SNP) (endothelium-independent). They found that Mas^−/−^ mice showed a marked decrease in the vasodilatory response to Ach and a slight increase in response to SNP, compared to control (Mas^+/+^) mice, suggesting that endothelium-dependent vascular reactivity was impaired in Mas^−/−^ mice [73]. Also, in a model of atherosclerosis using mice ApoE-KO or double KO also for Mas, it was found that Ang 1-7 mediated endothelial-dependent vasorelaxation specifically through the Mas receptor [72]. This feature was already proved by Langeveld and coworkers, who reported an improvement of endothelium-dependent relaxation in aortic rings after Ang 1–7 treatment in a rat stenting model, while no differences were detected for endothelial-independent relaxation [90]. Similarly, in a model of SHRSP rats overexpressing vascular ACE2, it was also reported an improved aortic endothelial function by means of a stronger relaxation response to the endothelium-dependent vasodilator, carbachol, and in vivo to the direct administration of ACh into the arterial system, which also lead to a decrease in blood pressure, suggesting that these effects may be due to the local degradation of Ang II to Ang 1-7 [91].

Moreover, Langeveld found a strong decrease in neointimal thickness, neointimal area, and percentage stenosis after Ang 1–7 treatment [90]. Another study in normotensive rats reported that short-term infusion of Ang 1-7 (or its nonpeptide analog, AVE 0991) enhances the hypotensive vasodilatory effect of ACh administration and this event is blunted by pretreatment with L-NAME or A-779 infusion [92]. Previous evidence reported that Ang 1-7 reduced neointimal formation, suggesting that this peptide might be involved in attenuated vascular growth in vivo after vascular injury [93,94]. Ang 1-7 was also found to play an antithrombotic effect in hypertensive rats developing venous thrombosis: in this model, Ang 1-7 administration resulted in a reduction of the thrombus weight, acting via Mas receptor through the activity of NO/PGI_2_ [95] therefore modulating Ca^2+^ mediated contraction [68]. Activation of the Ang 1-7/MasR axis enhances the effect of AT1R antagonism improving vascular remodeling and function in hypertensive rats [56]. The protective role of Ang 1-7 in vascular remodeling was also explored in a model of aortic aneurysm (AA) [66,96,97]: Ma and coworkers reported that AVE0991 (a nonpeptide Ang 1-7 mimic) and Ang 1-7 attenuate the formation of abdominal AA induced by Ang II in ApoE-KO mice, reduce abdominal aorta remodeling, pro-inflammatory cytokines release and oxidative stress through the suppression of pP38 and pERK1/2 signaling pathway [66]. Also in thoracic AA development, Ang 1-7 counteracts the pro-oxidative, inflammatory, vascular remodeling effect of Ang II [97].

Natriuretic peptides, particularly the atrial natriuretic peptide (ANP), are also known to have important vasodilatory and anti-hypertensive properties [98]. Recent evidence reported a strong protective effect of a novel dual-acting peptide (DAP) against vascular damage: this peptide, combining the Ang 1-7 sequence with part of the brain natriuretic peptide (BNP) and of ANP, enhances the cardiovascular protection provided by the single elements, acting via the co-activation of Mas and particulate guanylyl cyclase-A receptor, reducing H_2_O_2_-induced ROS generation, preventing vascular hypertrophy, attenuating intracellular calcium levels (and therefore, contraction) in VSMC and activating PI3K/AKT/eNOS pathway in ECs [99,100].

Ang 1-9, produced by ACE2 from Ang I, exerts a protective effect in vascular damage as well.

Continuous administration of Ang 1-9 in hypertensive rats (subjected to Ang II infusion or renal artery clipping) was found to reduce blood pressure and, in cultured rat cardiac fibroblasts, it reduced Ang II-induced fibroblast proliferation and ECM component deposition [77]. Moreover, in arteries from the same model, Ang II-treated rats showed reduced vasodilation in response to Ach, while Ang 1-9 co-infusion prevented this effect [77]. Using thoracic aortic rings from Sprague Dawley rats with an intact endothelium, pre-contracted with epinephrine, Ang 1-9 was found to play a significant vasodilatory role [77].

Moreover, Ang 1-9 reduces blood pressure and vascular remodeling in SHR lowering media thickness, reducing VSMC proliferation, and increasing ACE2 expression and Ang II circulating levels [101]. Moreover, through the AT2R, it also induces FoxO1 activation via Akt [101].

### 2.3. Organ Damage

According to what has been said so far, it seems clear that the ACE2/Ang 1-7/MasR axis plays an important role in cardiovascular prevention and protection from organ remodeling. Indeed, several studies reported the key role of ACE2 in diabetes [33], hepatic [38], lung inflammation [43], cardiac and renal function, vascular contractility, and endothelial function [34,35,36,37,39,40]. Also, Ang 1-7 plays a protective role in diabetes, vascular prevention, coagulation cascade, cardiac and renal damage [41,54] and pulmonary fibrosis [53]. Upregulation of the ACE2/Ang1-7/MasR axis also improved bone metabolism, mineralization, and mass and reduced RANKL expression which can be increased by Ang II stimulation [55]. A growing number of studies are also evidencing the anti-hypertensive and anti-remodeling role of Ang 1-9 in vascular, renal, and cardiac function [59]. Below, we focused on the evidence supporting the beneficial role of the ACE2/Ang1-7/MasR and Ang 1-9 in cardiac and renal function and remodeling.

#### 2.3.1. Heart

ACE2 expression has a crucial role in heart function since its loss is associated with an impaired contractile response, increased Ang II levels, and induced hypoxia-regulated gene expression profile [34]. ACE2 deficiency led to increased levels of Ang II that caused the development of cardiomyopathy, impaired left-ventricular function, cardiac hypertrophy, increase in the oxidative stress and neutrophils infiltration through the activation of PI3Kγ, a downstream mediator of the AT1R [35]. A comprehensive review from Patel [54] reported the effect of ACE2 on heart failure, concluding that clinical and experimental studies support the importance of ACE2/Ang 1-7 in this context and that enhancing this pathway might play a beneficial role in preventing heart disease [54].

Post-translational modifications such as glycosylation are processes naturally occurring in cell biology and physiology. Exploring the role of ACE2 glycosylation on Ang 1-9, Ang 1-7, and MasR in cardiomyocytes from patients with type 2 diabetes (under poor or good glycemic control), it was found that hearts from diabetic patients presented higher levels of glycosylated ACE2 which is related with poor glycemic control and an increase in cardiac fibrosis and remodeling and lower levels of Ang 1-7, Ang 1-9 and MasR, therefore blunting the effect of RAAS inhibitors. On the contrary, good glycemic control improved their effect ameliorating cardiac remodeling and enhancing the good branch of RAAS [102]. This is in line with the absence of cardiac remodeling observed in BS/GS patients [19]: indeed, they present low levels of glycosylated ACE2, probably due to their metabolic alkalosis and likely impairing the cellular glycosylation process [29].

Ang 1-7 receptor Mas plays an important role in cardiac function. Indeed, Mas deficiency is associated with a decreased fractional shortening, posterior wall thickness in systole and left ventricle end-diastolic dimension, and a higher left ventricle end-systolic dimension together with a lower global ventricular function and a high coronary perfusion pressure [103]. The beneficial role of Ang 1-7 in the heart has been widely described [104,105,106]. Chronic infusion of Ang 1-7 in a rat model for heart failure has been shown to preserve cardiac function, coronary perfusion, and aortic endothelial function [104], reduce cardiac hypertrophy [107], and strongly reduce Ang II levels in the heart of control Ang 1-7 infused rats [108]. Moreover, Ang 1-7 strongly prevents cardiac remodeling induced by Ang II [109].

Continuous administration of Ang 1-9 strongly ameliorates cardiac remodeling in terms of hypertrophy [110], left ventricular shortening, and ejection fraction [77]. Moreover, through the AT2R and activation of the Akt pathway, it improves in vitro cardiomyocyte survival, ex vivo reduced infarct size and left ventricular function in isolated perfused hearts underwent ischemia/reperfusion protocol, and in vivo reduced fibrosis and myocardial damage after induced myocardial infarction via left anterior descending artery ligation [111]. Previously, in the same in vivo model, also ACE2 was found to be an important protective agent in cardiac fibrosis, reducing ROS production and metalloproteinase activity compared to ACE2-deficient hearts, that instead presented also higher expression of the inflammatory markers and enhanced phosphorylation of the ERK1/2 and JNK1/2 signaling pathways [112]. Evaluating the production of Ang 1-9 and Ang 1-7 and their role in heart tissues, Jackman and coworkers found that these two peptides can burst the local effect of kinins enhancing NO and arachidonic acid release [113]. Moreover, an in vitro study using neonatal rat cardiomyocyte cell lines and adult primary rabbit cardiomyocytes transduced with adenoviral vectors that selectively over-express Ang 1-7 or Ang 1-9 reported inhibition of cardiomyocyte hypertrophy via Mas and AT2R, respectively [114].

#### 2.3.2. Kidney

The close relationship between the kidney and RAAS is known and involves the regulation of blood pressure, fluid homeostasis, and electrolyte balance. Also in the kidney, Ang II induces oxidative stress [115], organ remodeling, and dysfunction, therefore several studies also aimed at investigating the counterregulatory arm of the RAAS. ACE2 is widely expressed in the kidney [116,117] and an altered distribution of ACE2 is related to kidney disease [118].

ACE2-KO mice display an overactivation of NADPH oxidase due to Ang II-mediated activation of the AT1 receptor, which is related to increased production of ROS and H_2_O_2_ in the kidney, while scavenging systems were not changing between ACE2 KO and control mice [119]. The most abundant NADPH oxidase in the kidney is Nox4 [115], and it was found upregulated in ACE2 KO mice as well [119]. Moreover, Ang 1-7 was found to be increased in the kidney but not in the plasma of ACE2 KO mice [119]. Administration of rhACE2 in ApoE-KO mice infused with Ang II, ameliorates blood pressure and kidney remodeling increasing Ang 1-7 levels and reducing fibrosis through the downregulation of TGF-β1 and mTOR/ERK1/2 signaling [120]. ACE2 overactivation with Xanthenone improves kidney function, enhances anti-oxidant mechanisms, blunts inflammatory response, downregulates Ang II, and ameliorates tubular necrosis, leukocyte infiltration, cast formation, and glomerulonephrosis in a mice model of gentamicin-induced renal injury [121] and also in nephrotoxicity induced by calcineurin inhibitor tacrolimus [122].

An ex vivo study performed in the thick ascending loop (TAL) of rats explored the role of Ang 1-7 on oxidative stress and sodium transport, showing that Ang 1-7 via activation of Mas, decreases the transport-dependent oxygen consumption (VO_2_), increases NO levels [123]. Ang 1-7 subcutaneous administration in Akita mice (a well-known model of type 1 diabetes, spontaneously hypertensive, presenting an important renal remodeling and increased oxidative stress), resulted in decreased blood pressure, reduced ROS generation, NADPH oxidase activity, and Nox4 expression together with a lower expression of HO-1 and Nrf2 in the kidney of treated mice [124]. Moreover, Ang 1-7 markedly suppressed renal remodeling observed in Akita mice, reducing the expression of pro-fibrotic markers as collagen IV and TGF-β1 and ameliorating glomerular hyperfiltration, albuminuria, and renal hypertrophy [124]. Ang 1-7, via MasR activation, improves kidney injury in a model of obstructive nephropathy reducing apoptotic and fibrotic processes through the downregulation of the TGF-β1/Smad pathway, suppression of AT1R expression and ROS generation and recovery of G2/M cell cycle arrest [125]. The beneficial effect of Ang 1-7 was further investigated in a model of high fat-induced lipid metabolic disorders where it ameliorates renal remodeling by downregulation of the LDLr-SREBP2-SCAP pathway [126].

ANP plays an important role in sodium reabsorption favoring natriuresis and diuresis and maintaining cardiovascular homeostasis [98]. Also, renal infusion of Ang 1-7 induced natriuresis and diuresis via intrarenal Mas and AT1R, since their inhibition impaired also sodium and water excretion [127].

Combining Ang 1-7 with natriuretic peptides, Ghatage and coworkers created a dual activating peptide (DAP) that, activating both Mas and pGCA receptor, plays a beneficial role in cultured renal tubular NRK-52E epithelial cells, by decreasing the pro-oxidative and pro-inflammatory environment induced by H_2_O_2_ [100].

A strong link between congestive heart failure (CHF) and kidney function has been already described [128]. Exploring the role of Ang 1-7 in this context, it was found that chronic infusion of Ang 1-7 (or AVE0991) increases urinary sodium and potassium excretion, urinary cGMP, and reduced serum creatinine and aldosterone levels in rats who underwent placement of aortocaval fistula as a model of CHF compared to controls, evidencing the important role of the heptapeptide in CHF kidney function [107].

## 3. Conclusions

The first studies on the counterregulatory branch of RAAS were performed soon after the discovery of ACE2, elucidating the important role of this molecule in several pathophysiological pictures, regulating the local levels of Ang II and therefore blunting its deleterious effects in the cardiovascular system. A growing body of evidence indicates that Ang 1-7 and Ang 1-9 are strongly associated with a beneficial effect on cardiovascular inflammation and remodeling through the activation of Mas and AT2 receptors, respectively, improving heart and kidney function, and reducing oxidative stress and fibrosis. The results of the studies that have explored the role of the ACE2/Ang1-7/MasR/Ang 1-9 axis in clinical, cellular, and tissue settings obtained so far opened avenues for identifying further beneficial effects of the counter-regulatory arm of the RAAS on the clinical ground. Given the importance of these protective effects in a wide range of clinical scenarios, these roads must be run across.

## Data Availability

Not applicable.
